# A multilevel analysis of individual and contextual factors associated with the practice of safe disposal of children’s faeces in sub-Saharan Africa

**DOI:** 10.1371/journal.pone.0254774

**Published:** 2021-08-02

**Authors:** Abdul-Aziz Seidu, Bright Opoku Ahinkorah, Kwaku Kissah-Korsah, Ebenezer Agbaglo, Louis Kobina Dadzie, Edward Kwabena Ameyaw, Eugene Budu, John Elvis Hagan

**Affiliations:** 1 Department of Population and Health, University of Cape Coast, Cape Coast, Ghana; 2 College of Public Health, Medical and Veterinary Sciences, James Cook University, Townsville, Queensland, Australia; 3 The Australian Centre for Public and Population Health Research (ACPPHR), Faculty of Health, University of Technology Sydney, Ultimo, Australia; 4 Department of English, University of Cape Coast, Cape Coast, Ghana; 5 Neurocognition and Action-Biomechanics-Research Group, Faculty of Psychology and Sport Sciences, Bielefeld University, Bielefeld, Germany; 6 Department of Health, Physical Education, and Recreation, University of Cape Coast, Cape Coast, Ghana; University of Western Australia, AUSTRALIA

## Abstract

**Background:**

Over the years, sanitation programs over the world have focused more on household sanitation, with limited attention towards the disposal of children’s stools. This lack of attention could be due to the misconception that children’s stools are harmless. The current study examined the individual and contextual predictors of safe disposal of children’s faeces among women in sub-Saharan Africa (SSA).

**Methods:**

The study used secondary data involving 128,096 mother-child pairs of under-five children from the current Demographic and Health Surveys (DHS) in 15 sub-Saharan African countries from 2015 to 2018. Multilevel logistic analysis was used to assess the individual and contextual factors associated with the practice of safe disposal of children’s faeces. We presented the results as adjusted odds ratios (aOR) at a statistical significance of p< 0.05.

**Results:**

The results show that 58.73% (57.79–59.68) of childbearing women in the 15 countries in SSA included in our study safely disposed off their children’s stools. This varied from as high as 85.90% (84.57–87.14) in Rwanda to as low as 26.38% (24.01–28.91) in Chad. At the individual level, the practice of safe disposal of children’s stools was more likely to occur among children aged 1, compared to those aged 0 [aOR = 1.74; 95% CI: 1.68–1.80] and those with diarrhoea compared to those without diarrhoea [aOR = 1.17, 95% CI: 1.13–1.21]. Mothers with primary level of education [aOR = 1.42, 95% CI: 1.30–1.5], those aged 35–39 [aOR = 1.20, 95% CI: 1.12–1.28], and those exposed to radio [aOR = 1.23, 95% CI: 1.20–1.27] were more likely to practice safe disposal of children’s stools. Conversely, the odds of safe disposal of children’s stool were lower among mothers who were married [aOR = 0.74, 95% CI: 0.69–0.80] and those who belonged to the Traditional African Religion [aOR = 0.64, 95% CI: 0.51–0.80]. With the contextual factors, women with improved water [aOR = 1.13, 95% CI: 1.10–1.16] and improved toilet facility [aOR = 5.75 95% CI: 5.55–5.95] had higher odds of safe disposal of children’s stool. On the other hand, mothers who lived in households with 5 or more children [aOR = 0.89, 95% CI: 0.86–0.93], those in rural areas [aOR = 0.86, 95% CI: 0.82–0.89], and those who lived in Central Africa [aOR = 0.19, 95% CI: 0.18–0.21] were less likely to practice safe disposal of children’s stools.

**Conclusion:**

The findings indicate that between- and within-country contextual variations and commonalities need to be acknowledged in designing interventions to enhance safe disposal of children’s faeces. Audio-visual education on safe faecal disposal among rural women and large households can help enhance safe disposal. In light of the strong association between safe stool disposal and improved latrine use in SSA, governments need to develop feasible and cost-effective strategies to increase the number of households with access to improved toilet facilities.

## Background

Improved sanitation has been an important component of the global agenda aimed at promoting public health, as evident in Sustainable Development Goal (SDG) 6, which aims at ensuring that everyone gets access to water and sanitation [1, 2]. Inadequate sanitation leads to the transmission of pathogens through faeces and urine, which cause diseases such as cholera, diarrhoea, dysentery, hepatitis A, typhoid, and polio [3, 4]. It can also lead to active trachoma, some soil-transmitted helminth infections, and schistosomiasis [5, 6]. These are often transmitted by four main groups of pathogenic hazards (bacteria, viruses, protozoa, and helminths) [5, 7]. A report by WHO/UNICEF [5] reveals that, globally, about 2.3 billion people do not have access to improved sanitation. The same report estimates that basic sanitation is accessible to 68% of the world’s population, with as low as 28% of the people in sub-Saharan Africa (SSA) having access to basic sanitation. Globally, 775,000 people died prematurely as a result of poor sanitation in 2017 [8]. This estimate accounted for about 5% of total deaths in low- and middle-income countries, which is far above the global average of 1.4% [9].

Over the years, sanitation programs over the world have focused more on household sanitation, with limited attention towards the disposal of children’s stools. This lack of attention could be due to the misconception that children’s stools are harmless [10, 11]. Compared to adults, children, through some childhood behaviours, are more likely to come into contact with faecal pathogens [12]. For instance, when children play or crawl on the ground, they do not only get their fingers contaminated, but also put pica or fomite into their mouths [12]. Again, children may get sick more often and, thus, have more pathogens in their faeces. Additionally, they defecate in/around the household where other children could come into contact with faeces. This foregrounds the need for safe disposal of children’s faeces.

Modes of safe disposal of children’s stool include putting the faecal matter into a toilet/latrine, burying the matter, and children defecating in latrines. Alternatively, putting the stool in a drain or ditch, throwing it in the garbage, or throwing it in the open is considered an unsafe practice [13]. Recently, other forms of safe disposal of children’s stool have been considered to include the use of flush/pour-flush toilet, flush to piped sewer system, flush to septic tank, compositing toilet, and using container-based sanitation (refers to a system where toilets collect excreta directly in sealable, removable containers). On the other hand, unsafe disposals include the use of a bucket or other container for the retention of faeces, hanging toilet/hanging latrine, and defecating in the bush. Hence, children should be encouraged to use latrines and child faeces should be disposed of in a latrine [14].

For every society, safe disposal of children’s stool comes with some individual/maternal and contextual/household factors. Hence, their exploration could be beneficial to effective sanitation programs aimed at promoting safe disposal of children’s stool. Previous studies in India [14, 15], Bangladesh [16], Nigeria [17], Burkina Faso [18], Ethiopia [19], and Malawi [20] have identified several individual (e.g., age, media exposure, marital status, access to improve water, toilet facilities, age, and sex of child), and contextual (e.g., head of household head, wealth index, place of residence, region, number of people in a household) factors as predictors of safe disposal of children’s stool [20]. However, only few studies have used nationally-representative datasets of a number of sub-Saharan African countries to examine the predictors of safe disposal of children’s stool in the sub-Saharan region. Hence, gathering empirical evidence is essential, considering the challenges with sanitation practices in a number of sub-Saharan African countries [8]. Moreover, in examining the predictors of safe disposal of children’s stool, only few studies [19–22] used nationally-representative surveys and multilevel modelling [19, 20, 23] to better account for the hierarchical data structure of the surveys. To the best of our knowledge, there has not been any multi-country study on the predictors of safe stool disposal. This study, therefore, is a cross-country assessment of predicators for safe stool disposal in SSA, taking into account both individual and contextual predictors using multilevel modelling.

## Materials and methods

### Data source

Data for this study were obtained from current Demographic and Health Surveys (DHS) conducted between 2015 and 2019 in 15 sub-Saharan African countries (Table 1). The 15 countries were those that had recent DHS datasets and all the variables of interest in the study in their datasets. DHS is a nationwide survey undertaken across low- and middle-income countries every five-year period [24]. The survey is representative of each of these countries and targets core maternal and child health indicators such as unintended pregnancy, contraceptive use, skilled birth attendance, immunisation among under-fives, intimate partner violence, and issues regarding men’s health such as tobacco and contraceptive use. Children’s files (Kids Recode–KR files) were used for our study.

**Table 1:** Description of study sample and prevalence of safe stool disposal.

Survey country	Survey year	Sample^a^	Sample^b^	Sample^c^	Prevalence of safe stool disposal
1. Angola	2015–16	14,322	9,566	9,441	32.69
2. Benin	2018	13,589	8,452	8,302	34.13
3. Burundi	2016–17	13,192	8,427	8,391	77.04
4. Cameroon	2018	11,732	10,006	9,510	73.26
5. Chad	2014–15	18,623	16,246	15,593	26.38
6. Ethiopia	2016	10,641	6,272	6,188	38.62
7. Guinea	2018	7,951	4,357	4,274	56.89
8. Malawi	2015–16	17,286	8,941	8,857	84.67
9. Mali	2018	9,940	6,163	6,112	63.75
10. Nigeria	2018	33,924	19,730	19,458	56.95
11. Rwanda	2014–15	7,856	7,315	7,179	85.90
12. Senegal	2017	12,185	7,213	6,926	58.28
13. Uganda	2016	15,522	9,064	8,786	79.85
14. Zambia	2018	9,959	5,578	5,440	77.81
15. Zimbabwe	2015	6,132	3,796	3,639	75.75
**All countries**	–	202,858	131,126	128,096	58.73

^a^Sample size at design

^b^Women with complete information on children’s stool disposal

^c^Women with complete information on all variables of interest

In selecting the sample for each survey, stratified dual-stage sampling approach was employed. The first step of this sampling approach involved the selection of clusters (i.e., enumeration areas [EAs]), followed by systematic household sampling within the selected EAs. The sample size in the current study consisted of 128,096 mother-child pairs of under-five children with complete information on all the variables of interest. The respondents were mothers. Table 1 provides a description of the study sample. A detailed methodology of the DHS procedures has been discussed extensively elsewhere [24]. The dataset is freely available at www.measuredhs.com. We followed the “Strengthening the Reporting of Observational Studies in Epidemiology” (STROBE) statement in conducting this study and writing the manuscript (S1 Table).

### Study variables

#### Outcome variable

The outcome variable was safe disposal of children’s stool, captured as “safe” or “unsafe”. The variable was derived from the question “The last time [Name] passed stools, what was done to dispose of the stools?” The responses included “child used the toilet or latrine”, “put/rinsed into toilet or latrine,” “put/rinsed into drain/ditch,” “thrown into the garbage”, “buried”, “left in the open”, and “other”. Following the WHO’s [11] definition of safe and unsafe stool disposal, and previous studies [12, 15–17, 19, 25–28], these responses were recoded as follows: “Child used toilet or latrine”, buried and “put/rinsed into toilet or latrine” were combined and coded as “safe disposal of child stool” (coded as ‘1’) whereas the others were coded as “unsafe disposal of child stool” (coded as ‘0’). Hence, in this study, safe disposal of stool includes “child used toilet or latrine”, buried and “put/rinsed into toilet or latrine” while “thrown into the garbage,” “left in the open,” and “other” were considered as unsafe disposal of child stool.

#### Independent variables

Selected variables were included based on their association with safe disposal of children’s stool in previous studies [12, 15–17, 19, 20, 25–28] and availability of variables in the data. Seventeen explanatory variables were included in the study and were grouped into two:

1. Individual level factors (child and maternal variables): sex of child (male, female), age of the child (0,1,2,3,4), and child’s experience diarrhoea in the last two weeks (yes, no). Mother’s age (15–19, 20–24, 25–29, 30–34, 35–39, 40–44, 40+), mother’s educational level (no education, primary, secondary, and higher), marital status (never married, married, cohabiting, widowed/divorced/separated), religion (Christianity, Islam, Traditional, no religion, Other), and mother’s exposure to mass media (newspaper, television, and radio) which were captured as Yes or No.
2. Contextual level factors: number of people in household (less 5, 5 or more), sex of household head (male, female), household’s wealth status (poorest, poorer, middle, richer, richest), place of residence (urban, rural) and sub-region. Sub-region was categorised as Central Africa (Angola, Chad), West Africa (Benin, Guinea, Mali, Nigeria, Senegal), East Africa (Burundi, Cameroon, Ethiopia, Malawi, Uganda, Rwanda) and Southern Africa (Zambia, Zimbabwe). Wealth index (poorest, poorer, middle, richer and richest), in the DHS, is a composite measure computed by combining data on a household’s ownership of carefully identified assets including television, bicycle, materials used for house construction, sanitation facilities and type of water access. Principal component analysis was used to transform these variables into wealth index by placing individual households on a continuous measure of relative wealth. The DHS segregates households into five wealth quintiles; poorest, poorer, middle, richer and richest. Additionally, the toilet facility and source of drinking water were categorized into “improved” and “unimproved” [29].

### Data analyses

The datasets were pooled by recoding the variables in the respective countries and using the ‘append’ command to pool them together as a single file. Data were analysed at the univariate, bivariate, and multivariate levels. Prevalence of safe disposal of children’s faeces and socio-demographic characteristics were described using frequencies and percentages. At the bivariate level, a chi-square test was carried out between the independent and dependent variable at p<0.05. We selected all the variables that showed statistical significance for the multilevel binary logistic regression model which was used due to the hierarchical nature of the data [30, 31]. A two-level multilevel binary logistic regression analysis was done to assess the individual and contextual factors associated with disposal of children stools. Per the two-level modelling, women were nested within clusters to account for the variance in primary sampling units (PSUs). Clusters were regarded as random effect to take care of the unexplained variability at the contextual level. We fitted four models. First, we fitted the empty model, Model 0, that had no predictors (random intercept). This procedure was followed by Model 1, which contained only the individual level variables (child and maternal factors), Model 2 with only contextual level variables, and Model 3, with both individual and contextual level variables. For all models, we presented the adjusted odds ratios (aOR) and associated 95% confidence intervals.

For model comparison, we used the Akaike information criterion (AIC) test [32, 33]. Model adequacy was checked using the LR test. Using the variance inflation factor (VIF), the multicollinearity test showed that there was no evidence of collinearity among the independent variables (Mean VIF 1.24, Maximum VIF = 1.62, Minimum VIF = 1.03). The choice of reference categories was informed by previous studies [12, 15–17, 19, 20, 25–28] and practical significance. Sample weight was applied in all the analysis to correct for over- and under-sampling while the “svy” command was used to account for the complex survey design and generalizability of the findings. According to Hatt and Waters [34], pooling data can reveal broader results that are ‘‘often obscured by the noise of individual data sets.” To calculate the pooled values, an additional adjustment is needed to account for the variability in the number of individuals sampled in each country. This method was accomplished using the weighting factor 1/(A*n_c_ /n_t_), where A refers to the number of countries where a particular question was asked, n_c_ denotes the number of respondents for the country c, and n_t_ indicates the total number of respondents over all countries where that question was asked. All the analyses were carried out using Stata Version 14.2 for MacOS. Statistical significance was set at p < 0.05.

### Ethical approval

Ethical clearance for DHS surveys is usually obtained from the Ethics Committee called Inner City Fund Institutional Review Board (IRB) as well as Ethics Boards of partner organisations of the various countries such as the Ministries of Health. During the surveys, either written or verbal consent was provided by the targeted women. Since the data were not collected by the authors of this manuscript, permission was sought from MEASURE DHS website and access to the data was provided after our intent for the request was assessed and approved on 3rd April, 2019. The data is available on https://dhsprogram.com/data/available-datasets.cfm.

## Results

### Descriptive statistics on the prevalence of safe disposal of child stools in SSA

**Fig 1:**
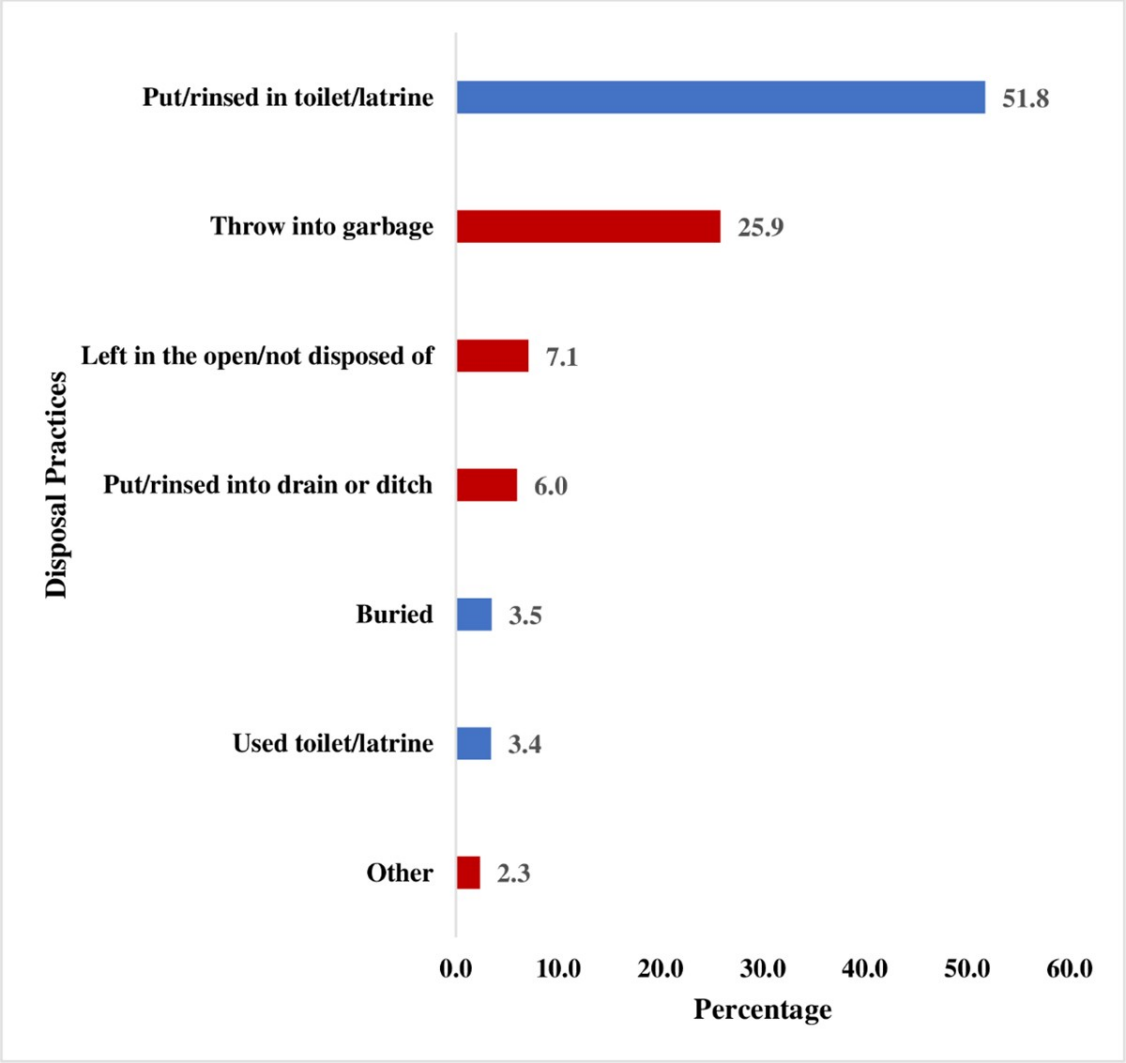
Prevalence of safe disposal of child stools by types of disposal in sub-Saharan Africa. Red: Unsafe, Blue: Safe.

Table 1 presents the results on safe disposal of children’s stools in the 15 countries in SSA included in this study. Overall, 58.73% (57.79–59.68) of childbearing women in the 15 countries in SSA included in our study safely disposed of their children’s stools. This varied from as high as 85.90% (84.57–87.14) in Rwanda to as low as 26.38% (24.01–28.91) in Chad (Table 1). Most of the mothers who safely disposed of children’s stool put/rinse it in a toilet or latrine (51.8%) while for a few of them, the children used toilet/latrine (3.4%). In terms of those who practised unsafe disposal of children’s stool, the majority of them threw the faeces into garbage (25.9%) while a few of them disposed of the stool using other means (2.3%) (Fig 1).

### Bivariate analysis on the prevalence of safe child’s stool disposal across independent variables among women in SSA

Table 2 shows the results on the prevalence of safe disposal of children’s stool among women in SSA across the explanatory variables. Apart from sex of children, all the other explanatory variables had significant associations with safe disposal of children’s stools at 95% confidence interval. In terms of the individual level factors, safe disposal was high for children aged one year (64.1; 95% CI = 64.1–66.2), children who experienced diarrhoea (62.1; 95% CI = 60-7-63.4), and children whose mothers had no formal education (49.2; 95% CI = 47.9–50.6). Additionally, high prevalence of safe disposal of stool was found among mothers aged 35–39 (60.2; 95% CI = 58.7–61.6), widowed/divorced/separated (66.9; 95% CI = 64.7–69.0), Christians (61.9; 95% CI = 60.7–63.0), and those exposed to newspaper (68.4; 95% CI = 66.9–70.0), television (64.3; 95% CI = 63.1–65.5), and radio (64.9; 95% CI = 64.0–65.9). For contextual factors, the prevalence of safe disposal of children’s stool was high among women within the richest wealth quintile (70.0; 95% CI = 68.5–71.6), those in female-headed households (61.0; 95% CI = 59.8–62.4), those with improved access to water (62.9; 95% CI = 61.8–63.9), those with improved toilet facility (72.5; 95% CI = 71.7–73.3), those in households with less than 5 members (62.1; 95% CI = 60.9–63.2), those in urban areas (63.9; 95% CI = 62.4–65.5), and mothers in Central Africa (71.3; 95% CI = 69.4–73.2).

**Table 2:** Prevalence of safe children’s stool disposal across independent variables among women in SSA.

Variables	Sample (128,096)				
	Weighted N	Weighted %	Safe disposal(%)	95% CI	
**Individual level factors**					
**Childs age (p<0.001)**					
0	38,993	30.4	54.2	53.2	55.1
1	36,329	28.4	65.2	64.1	66.2
2	13,605	10.6	55.6	54.2	57.1
3	19,979	15.6	58.0	56.7	59.3
4	19,190	15.0	58.8	57.3	60.1
**Sex of baby (p = 0.657)**					
Male	64367	50.3	58.8	57.7	59.8
Female	63729	49.7	58.7	57.7	59.7
**Child experience diarrhoea (p<0.001)**					
No	104,228	81.4	58.0	57.0	58.9
Yes	23,868	18.6	62.1	60.7	63.4
**Education(p<0.001)**					
No education	54,105	42.2	49.2	47.9	50.6
Primary	44,312	34.6	66.1	64.9	67.2
Secondary	25,694	20.1	65.3	64.0	66.6
Higher	3,985	3.1	63.7	60.7	66.6
**Age of mother (p<0.001)**					
15–19	8,789	6.9	55.1	53.4	56.8
20–24	30,250	23.6	59.2	58.0	60.4
25–29	36,353	28.4	57.8	56.6	58.9
30–34	27,111	21.2	59.9	58.6	61.2
35–39	17,181	13.4	60.2	58.7	61.6
40+	8,413	6.6	58.3	56.1	60.5
**Marital status (p<0.001)**					
Never married	5,077	4.0	63.5	61.3	65.7
Married	97,620	76.2	58.3	57.3	59.4
Living with partner	19,206	15.0	56.8	54.8	58.7
Widowed/divorced/separated	6,192	4.8	66.9	64.7	69.0
**Religion (p<0.001)**					
Traditionalist	1,704	1.3	39.3	34.1	44.8
Christianity	74,699	58.1	61.9	60.7	63.0
Islam	48,598	37.9	55.7	54.2	57.3
No religion	2,506	2.0	36.2	32.4	40.2
Other	588	0.5	60.4	52.2	68.0
**Exposure to Newspaper(p<0.001)**					
No	112,953	88.2	57.4	56.4	58.4
Yes	15,143	11.8	68.4	66.9	70.0
**Exposure to television (p<0.001)**					
No	85,129	66.5	55.9	54.8	57.1
Yes	42,967	33.5	64.3	63.1	65.5
**Exposure to Radio (p<0.001)**					
No	62,685	48.9	52.2	51.0	53.5
Yes	65,411	51.1	64.9	64.0	65.9
**Contextual level factors**					
**Household wealth status (p<0.001)**					
Poorest	29,947	23.4	48.8	47.2	50.4
Poorer	28,623	23.4	56.3	54.8	57.7
Middle	26,210	20.5	60.6	59.1	62.0
Richer	23,337	18.2	62.7	61.1	64.2
Richest	19,978	15.6	70.0	68.5	71.6
**Household head sex (p<0.001)**					
Male	106,347	83.0	58.3	57.3	59.2
Female	21,749	17.0	61.0	59.8	62.4
**Access to water (p<0.001)**					
Unimproved	52,992	41.4	52.8	51.5	54.3
Improved	75,104	58.6	62.9	61.8	63.9
**Type of Toilet facility (p<0.001)**					
Unimproved	35,738	27.9	23.2	22.1	24.3
Improved	92,358	72.1	72.5	71.7	73.3
**Number of people in household(p<0.001)**					
Less than 5	30,262	23.6	62.1	60.9	63.2
5 or more	97,834	76.4	57.7	56.7	58.7
**Residence(p<0.001)**					
Urban	35,573	27.8	63.9	62.4	65.5
Rural	92,523	72.2	56.7	55.6	57.9
**Sub-region (p<0.001)**					
Central Africa	24,234	18.9	71.3	69.4	73.2
West Africa	45,091	35.2	46.1	44.6	47.6
East Africa	49,574	38.7	25.5	24.3	26.8
Southern Africa	9,197	7.2	23.0	21.3	24.9

*P-values are from chi-square test

### Multilevel logistic regression results

#### Fixed effects results

Table 3 shows the results of the multilevel logistic regression analysis. The final model (Model 3) is the complete model that shows how the individual and contextual level factors interact to influence safe disposal of children’s stools in SSA. The results indicate that with the individual level factors, the practice of safe disposal of children’s stools was more likely to occur among children aged 1, compared to those aged 0 (aOR = 1.74; 95% CI: 1.68–1.80) and those with diarrhoea, compared to those without diarrhoea (aOR = 1.17, 95% CI: 1.13–1.21). Mothers with primary level of education were more likely to practise safe disposal of children’s stools compared to those with higher education (aOR = 1.42, 95% CI: 1.30–1.5), those aged 35–39 compared to those aged 15–19 (aOR = 1.20, 95% CI: 1.12–1.28), and those exposed to radio compared to those who were not exposed (aOR = 1.23, 95% CI: 1.20–1.27). Conversely, the odds of safe disposal children’s stool were lower among mothers who were married compared to those who were never married (aOR = 0.74, 95% CI: 0.69–0.80), and Traditionalists compared to those who belonged to other religions (aOR = 0.64, 95% CI: 0.51–0.80). With the contextual factors, women with improved water (aOR = 1.13, 95% CI: 1.10–1.16) and improved toilet facility (aOR = 5.75 95% CI: 5.55–5.95) had higher odds of safe disposal of children’s stool compared to those in households with unimproved water and toilet facility. Also, mothers who lived in households with 5 or more children (aOR = 0.89, 95% CI: 0.86–0.93), those in rural areas (aOR = 0.86, 95% CI: 0.82–0.89), and those who lived in Central Africa (aOR = 0.19, 95% CI: 0.18–0.21) were less likely to practise safe disposal of children’s stools, compared to those who lived in households with less than 5 people, urban residents, and those in Southern Africa.

**Table 3:** Predictors of safe child stool disposal practices among women in SSA.

	Model 0	Model 1	Model 2	Model 3
		AOR[95%CI]	AOR[95%CI]	AOR[95%CI]
**Fixed effects results**				
**Individual level factors**				
**Childs age**				
0		Ref		Ref
1		1.59***[1.54,1.65]		1.74***[1.68,1.80]
2		1.09***[1.05,1.14]		1.30***[1.24,1.36]
3		1.23***[1.18,1.27]		1.46***[1.40,1.52]
4		1.26***[1.22,1.31]		1.48***[1.42,1.55]
**Child experience Diarrhoea**				
No		Ref		Ref
Yes		1.17***[1.13,1.21]		1.17***[1.13,1.21]
**Education**				
No education		0.54***[0.50,0.59]		1.14**[1.04,1.25]
Primary		1.14** [1.05,1.24]		1.42***[1.30,1.55]
Secondary		1.07[0.98,1.16]		1.31***[1.20,1.42]
Higher		Ref		Ref
**Age of mother**				
15–19		Ref		Ref
20–24		1.15***[1.09,1.21]		1.10**[1.04,1.16]
25–29		1.15***[1.09,1.21]		1.09**[1.03,1.16]
30–34		1.29***[1.23,1.37]		1.18***[1.11,1.25]
35–39		1.31***[1.23,1.38]		1.20***[1.12,1.28]
40+		1.29***[1.21,1.38]		1.18***[1.10,1.28]
**Marital status**				
Never married		Ref		Ref
Married		0.973[0.91,1.04]		0.74***[0.69,0.80]
Living with partner		0.77*** [0.72,0.83]		0.83***[0.76,0.90]
Widowed/divorced/separated		1.23***[1.13,1.34]		1.02[0.93,1.12]
**Religion**				
Traditionalist		0.46***[0.37,0.56]		0.64***[0.51,0.80]
Christianity		0.89[0.74,1.06]		0.95[0.78,1.15]
Islam		0.76**[0.64,0.91]		1.13[0.93,1.37]
No religion		0.39***[0.32,0.48]		0.72**[0.57,0.89]
Other		Ref		Ref
**Exposure to Newspaper**				
No		Ref		Ref
Yes		1.12***[1.07,1.17]		0.99[0.95,1.05]
**Exposure to television**				
No		Ref		Ref
Yes		1.19***[1.16,1.23]		1.01[0.97,1.04]
**Exposure to Radio**				
No		Ref		Ref
Yes		1.56***[1.52,1.61]		1.23***[1.20,1.27]
**Contextual level factors**				
**Household wealth status**				
Poorest			0.83***[0.79,0.88]	0.85***[0.80,0.91]
Poorer			0.96[0.91,1.01]	0.96[0.91,1.02]
Middle			1.03[0.98,1.09]	1.03[0.97,1.08]
Richer			0.96 [0.92,1.01]	0.95[0.90,1.00]
Richest			Ref	Ref
**Household head sex**				
Male			0.90***[0.86,0.93]	0.97[0.93,1.01]
Female			Ref	Ref
**Type of water used in household**				
Unimproved			Ref	Ref
Improved			1.14*** [1.11,1.17]	1.13***[1.10,1.16]
**Type of Toilet facility**				
Unimproved			Ref	Ref
Improved			5.99***[5.79,6.20]	5.75***[5.55,5.95]
**Number of people in household**				
Less than 5			Ref	Ref
5 or more			0.93*** [0.90,0.96]	0.89***[0.86,0.93]
**Residence**				
Urban			Ref	Ref
Rural			0.83*** [0.80,0.86]	0.86***[0.82,0.89]
**Sub-region**				
Southern Africa			Ref	Ref
Central Africa			0.20***[0.19,0.21]	0.19***[0.18,0.21]
West Africa			0.34***[0.32,0.36]	0.33***[0.31,0.35]
East Africa			0.68***[0.64,0.72]	0.65***[0.61,0.69]
**Random effect results**				
**Parameter**				
Contextual-level variance(SE)	0.62(0.04)	1.10(0.06)	0.53(0.04)	0.53(0.04)
ICC	0.16	0.25	0.14	0.14
Log-likelihood	-85115.0	-80599.4	-69263.58	-68310.6
LR Test	*χ*^*2*^ = 3743.9,	*χ*^*2*^ = 4499.1,	*χ*^*2*^ = 2601.6,	*χ*^*2*^ = 2509.1
	p< 0.001	p< 0.001	p< 0.001	p< 0.001
AIC	170234.1	161248.8	138555.2	136695.3
N	128,096	128,096	128,096	128,096

Exponentiated coefficients; 95% confidence intervals in brackets

* *p* < 0.05

** *p* < 0.01

*** *p* < 0.001, Ref = Reference category; ICC = Intra-Class Correlation; AIC = Akaike’s Information Criterion; SE: Standard Error

Model 0 = The null model, a baseline model without any determinant variable

Model 1 = Individual level variables

Model 2 = Contextual level variables

Model 3 = The final model adjusted for individual and contextual level variables

#### Random effects results

With the random effects results, the complete model (Model 3), which included all the individual and contextual level factors, was considered as the best fit model for predicting the occurrence of safe disposal of children’s stool among women. This model explained 14% of the variation in safe disposal of children’s stool (ICC = 0.14). The percentage of variance explained at the empty model was 0.16. This figure increased to 0.25 in Model I but decreased to 0.14 in Models 2 and 3.

## Discussion

This study examined the influence of child, maternal, and contextual factors on safe disposal of children’s stools in SSA. Overall, 58.73% (57.79–59.68) of childbearing women practised safe disposal in the selected 15 countries in SSA and varied from 85.90% in Rwanda to 26.38% in Chad. When we aggregated, all regions (Central, Western, and Eastern) had lower odds of safe disposal of children’s stool disposal compared to Southern Africa. It is possible that countries within the other three sub-regions have some commonalities with latrine coverage and improved sanitation facilities. Provision of child faeces management tools will not be useful in improving children’s faeces disposal unless households have access to sanitation facilities to dispose of faeces. Compared with earlier reports, the prevalence observed in Rwanda is higher than the prevalence of several other SSA countries including Zambia (67%) [35], Kenya (70%) [36], Uganda (75%) [37], and Malawi (79–85.6%) [20, 38]. The high prevalence of safe disposal of children’s faeces noted in some countries such as Rwanda and Malawi possibly suggests that these countries may have specific community led total sanitation (CLTS) which is part of water and sanitation (WASH) programs. For example, Rwanda has been described as one of the countries in SSA with low open defecation [39]. The 2014 Integrated Household Living Conditions Survey revealed that 83% of households were using improved sanitation facilities. Various interventions have been implemented to achieve this goal. There are also various organisations, including UNICEF, that are currently working in 10 out of the 30 districts in Rwanda to promote sanitation. Similarly, WaterAid has worked with several communities in Rwanda and constructed a number of latrines and motivated the citizenry to avoid improper faeces disposal and open defecation [40]. Between 2010 and 2015, there were continued efforts in raising awareness and open defecation free campaigns, which sometimes include child stool disposal under WASH and open defecation programs [20]. Nonetheless, Pickering et al. [41] found, in their randomised trial in Malawi, that there were no differences in diarrhoea prevalence among the villages that received CLTS program and those that did not.

Children’s age and diarrheal status were two child factors that influenced safe disposal of children’s stools. Specifically, the practice of safe disposal of children’s stools was more likely to occur among age one children, compared to less-than-a-year children. Usually, mothers of older children are more likely to practise safe disposal of children’s stools, findings which corroborate previous studies in Cambodia [1], Bangladesh [16], and Nigeria [42]. The possible reason for the finding could be linked to the definition of safe disposal of children’s stool as “child used toilet or latrine” and “put/rinsed into toilet or latrine” [13]. Proper and effective use of latrine by a child is associated with age, with older children more likely to effectively and independently use latrine and avoid open defecation, compared to younger ones [1, 16, 42]. Again, faeces of young children look smaller, smell less, have fewer visible food residues, and are considered less harmful. Conversely, faeces of older children have bad smell and possess visible food residues that make them more disgusting or unpleasant, and are considered quite harmful [16]. These assertions also possibly explain why faecal matter of older children is disposed safely, as compared to their younger counterparts. Mothers with children who have diarrhoea were more likely to practise safe disposal of children’s stools, with evidence suggesting that diarrhoea is often associated with improper sanitation practices [43, 44]. Presumably, this linkage is because mothers, other family members, guests, and the wider community are less likely to consider stools as harmless [43, 44]. Evidence from Iraq indicates that children from households where faeces were disposed of in a latrine were less probable to experience diarrhoea [45]. It is, however, noteworthy that just having access to improved toilets does not guarantee utilisation of the toilet facility for children’s faeces disposal [12]. Hence, once the child has diarrhoea, practising safe disposal of children’s stools plays a key role in enhancing his/her recovery process and ensuring improvement in the health and wellbeing of the child. Behavioural interventions that inspire consistent proper utilisation of toilets by children at a younger age might be very impactful towards improving children’s positive or safe faeces disposal practices [46]. It is, however, noteworthy that the question on diarrhoea was about the last two weeks and the question on disposal was the last time the child defecated and as such, it is possible that these two variables are probably not measuring the same defecation.

On maternal factors, the practice of safe disposal of children’s stools was less likely to occur among Traditionalists, compared to women of other/unspecified religions. This finding could be due to differences in beliefs, as well as sociocultural and other doctrinal codes, between Traditionalist religion and the others [46, 47]. Despite this difference, other studies found no variation across religions [42, 48]. The reasons for these conflicting results might be the varied geographical locations where these studies were conducted and other methodological considerations. Safe disposal of children’s stools was high among widowed/divorced/separated women, those aged 35–39 as well as women aged 40 and above compared to those who had never married and women aged 15–19. The findings are consistent with findings of a study by Azage and Haile [19] conducted in Ethiopia on factors associated with safe child’s faeces disposal practices. The possibility is that divorced women may be more conscious of adverse implications of unsafe faecal disposal on child’s health based on their previous experiences [42]. Further, divorced women tend to be older and maternal/caregiver’s age is associated with safe child faeces disposal practices [42]. However, women who have never married are likely to be young. As a result, they may possess little experience and knowledge about the consequences of unsafe faecal disposal on children’s health.

Unlike previous studies that found no association between mothers’ education and safe disposal of child stools [20–22, 49], this present study revealed that mothers without formal education were more likely to practise safe disposal. This finding seems counter-intuitive; however, the absence of formal education does not imply the absence of health education or knowledge on personal hygiene. Consequently, our finding is plausible. However, this current finding calls for the need for further studies on the association between education and stool disposal. Mothers’ access to information through mass media (television and radio), and ANC attendance may enhance safe children’s faeces disposal practice. Several authors [19, 23, 42] have explained that women exposed to media and those who attend ANC are more likely to have information on positive attitudes and may value the importance of safe disposal of children’s stools. Other scholars have also postulated that mothers who have exposure to media and those who attend ANC are more exposed and in a better position to understand the causes of childhood illnesses and consider the practice of hygienic behaviours, such as safe disposal of children’s stool and other childcare practices (e.g., good grooming), as significant in protecting their children from illness [19, 50].

With contextual factors, low socio-economic status manifested in less utilisation of improved toilet facilities. Thus, being in poorest wealth quintile was associated with lower odds of safe disposal of child’s stool compared with being in richest wealth quintile. Previous studies have also found significant effects of high socio-economic status and standard of living on improved household sanitation practices [18, 51–53]. Several reasons may explain this finding, including access to better sanitation facilities (which, in this study, includes source of drinking water and toilet facilities) among rich women, which promote safe disposal of their children’s faeces [52, 53].

### Strength and limitations

The main strength of this study is the use of nationally representative data from 15 countries in SSA. With this data pool, it is possible to generalise the findings of this study to women with children under five years in all the countries that participated in the study. Notwithstanding, the research design was cross-sectional in nature; hence, causality cannot be claimed for the findings obtained. We also limited our study to countries with relatively recent DHS datasets. The outcome variable, disposal of a child stool, was collected based on reported practice rather than direct observation. Future research could use spot checks and structured observations rather than questionnaires. With these assessment approaches, less bias could be obtained [54]. There is also the possibility of social desirability and recall biases. However, recall bias is likely to be minimal since mothers were asked the question, “The last time (NAME) passed stools, what was done to dispose of the stools?”. Because secondary data were used, all variables that may impact the practice of safe disposal of children’s faeces were not comprehensively included in the analysis. For example, mothers’ perceptions and knowledge on the negative implications of children’s faeces were not added in the survey [19, 55]. Also, odds ratios are usually inflated when sample size for a variable is too minimal, such as in the case with variables like Islam religion, divorced women, among others. This error may possibly create model results that show high levels of significance for a variable, when it is actually due to small sample size of that variable and may not be a generalisable finding. Quite a large number of variables were included in our models and as such, some of the significant results may be due to chance. In addition, the categorisation may not be applicable in some high-income countries where disposal of diapers may be considered a safe practice. Despite these limitations, the current study highlights mothers’ safe disposal of children’s faeces in selected SSA countries and, thus, complements the growing body of empirical evidence related to proper sanitation practices associated with child and maternal health.

## Conclusion

The study has revealed a wide variation in safe disposal of children’s faeces across the participating countries. The findings underscore the need for promoting practical and context-based interventions aimed at encouraging the practice of safe disposal of children’s stool to take cognisance of these identified factors, especially in countries that recorded high unsafe faeces disposal rates. Between- and within-country contextual variations and commonalities need to be acknowledged in designing interventions to enhance safe disposal of children’s faeces. Behavioural change communication by adopting audio-visual education on safe faecal disposal among rural women and large households can help enhance safe disposal. Active engagement of community leaders and community health workers is also critical to identify misconceptions on children’s stool disposal. In the light of the strong association of safe stool disposal with improved latrine use in SSA, governments may have to develop feasible and cost-effective strategies to increase the number of households with access to improved toilet facilities. More studies are required to evaluate children’s defecation practices in many low- and middle-income countries, perhaps with more than one methodology. For instance, incorporating participatory observational techniques with qualitative methods could provide more elaborate, ecologically-valid, and precise findings.

## Supporting information

S1 Table STROBE 2007 (v4) statement—checklist of items that should be included in reports of *cross-sectional studies*. (DOCX)

## Abbreviations

ANC: Antenatal Care
AOR: Adjusted Odds Ratio
CI: Confidence Interval
DHS: Demographic and Health Survey
SSA: sub-Saharan Africa
SDG: Sustainable Development Goal
MLRM: Multilevel Logistic Regression Model
WHO: World Health Organisation
ICC: Intra-cluster Correlation Coefficient
AIC: Akaike’s Information Criterion
